# Recurrence of Statin-Induced Necrotizing Myopathy: A Learning Point

**DOI:** 10.7759/cureus.53945

**Published:** 2024-02-09

**Authors:** Mohamed Qasim Toorani, Amir Alvi

**Affiliations:** 1 Rheumatology, Diana Princess of Wales Hospital, Grimsby, GBR

**Keywords:** statin intolerant patients, statin- induced myositis, statin-associated myopathy, statin-induced myopathy, statin-induced autoimmune necrotizing myopathy

## Abstract

Statin-induced myopathy remains a significant adverse event associated with statin use. Insufficient literature exists studying the recurrence of statin-induced myopathy in patients who have been re-exposed to statins. In this case report, we present the case of an elderly woman who developed immune-mediated necrotizing myopathy secondary to simvastatin and eventually improved with statin cessation and corticosteroids. Two years after her initial presentation, she re-developed myopathy when she was prescribed atorvastatin in the community. This case highlights the importance of recognizing that statin-induced myopathy is likely a class effect and being wary of re-prescribing statins in this vulnerable population group.

## Introduction

Statins have been prescribed for primary and secondary prevention of atherosclerosis for decades. Unfortunately, statin-induced myopathy is the commonest cause of discontinuation of a statin in up to 15% of patients [1]. Insufficient literature exists studying the recurrence of statin-induced myopathy in patients who have been re-exposed to statins. In this case report, we present a woman who developed statin-induced necrotizing myopathy and, after a few years, re-developed the myopathy when she was prescribed another statin.

## Case presentation

An elderly woman with a history of non-insulin-dependent diabetes mellitus and learning difficulties presented to the hospital with a four-month history of unintentional weight loss and proximal muscle weakness. Her symptoms were gradually progressing and affecting her activities of daily living. Clinical examination revealed proximal muscle weakness in the shoulder and pelvic girdle groups of muscles, with power graded at 3/5 in both groups. Distal muscles were noted to have full strength. Cardiovascular, respiratory, and gastrointestinal examinations were unremarkable. Vital signs on admission included a heart rate of 98 beats per minute, a respiratory rate of 16 breaths per minute, a temperature of 37.1° C, a blood pressure of 115/62 mmHg, and oxygen saturations of 94% on room air. Blood tests were taken on admission (Table 1).﻿

**Table 1:** Blood test results on admission, with reference ranges.

Blood Test	Result	Reference range
Creatine kinase (CK)	3748 U/L	25-200 U/L
Haemoglobin	150 g/L	117-149 g/L
White cell count	8.5 x 10*9/L	4.3-11.2 x 10*9/L
Platelets	377 x 10*9L	150-400 x 10*9/L
C-reactive protein	1.7mg/L	0-5mg/L
Sodium	143 mmol/L	133-146 mmol/L
Potassium	4.4 mmol/L	3.5-5.3 mmol/L
Chloride	106 mmol/L	95-108 mmol/L
Urea	6.3 mmol/L	2.5-7.8 mmol/L
Creatinine	22 umol/L	45-84 umol/L
Estimated glomerular filtration rate	>90 mL/min	>90 mL/min
Bilirubin	28umol/L	0-21 umol/L
Alanine transaminase	181 U/L	0-33 U/L
Antinuclear antibody (ANA)	Negative	
Anti-neutrophil cytoplasmic antibody (ANCA)	Negative	
Anti-smooth muscle antibody	Negative	
Anti-liver kidney microsomal antibody	Negative	

Electromyography (EMG) revealed features suggestive of myositis. Magnetic resonance imaging (MRI) of the pelvic girdle and hips was done to further assess myositis. It revealed that there was a slight loss of joint space height in the hips with slightly increased short tau inversion recovery (STIR) signals within the adductor muscles and several of the gluteal muscles bilaterally, which was consistent with the history of a myopathy.

She had been on 20mg of simvastatin before her admission, which was stopped while in the hospital. A myositis antibody screen was sent, which was negative (Table 2).

**Table 2:** Myositis screen tested Anti EJ antibody: anti-glycyl tRNA synthetase, Anti-HMG-CoA reductase antibody: anti-3-hydroxy-3-methylglutaryl-CoA reductase antibody, Anti-Jo antibody: anti-histidyl tRNA synthetase, anti-Ku antibody: Ku70/Ku80 antibody, MDA5 antibody: melanoma differentiation-associated gene 5 antibody, Mi-2-Alpha antibody: nucleosome remodelling deacetylase alpha antibody, Mi-2-Beta antibody: nucleosome remodelling deacetylase beta antibody, NXP-2 antibody: anti-nuclear matrix protein 2 antibody, Anti-OJ antibody: anti-isoleucyl tRNA synthetase, Anti-PM-SCL 100 antibody: anti-polymyositis-scleroderma 100 antibody, Anti-PM-SCL 75 antibody: anti-polymyositis-scleroderma 75 antibody, Ro-52 Antibody: anti–Sjögren's-syndrome-related antigen A antibodies, SAE-1 antibody: anti-small ubiquitin-like modifier 1-activating enzyme antibody, anti-SRP antibody: anti-signal recognition particle antibody, TIF-Gamma antibody: transcription intermediary factor gamma antibody.

Antibody	Result
Anti-EJ Antibody	Negative
Anti-HMG-CoA Reductase Antibody	Negative
Anti-Jo Antibody	Negative
Anti-Ku Antibody	Negative
MDA5 Antibody	Negative
Mi-2-Alpha Antibody	Negative
Mi-2-Beta Antibody	Negative
NXP-2 Antibody	Negative
Anti-OJ Antibody	Negative
Anti-PM-SCL 100 Antibody	Negative
Anti-PM-SCL 75 Antibody	Negative
Ro-52 Antibody	Negative
SAE-1 Antibody	Negative
Anti-SRP Antibody	Negative
TIF-Gamma Antibody	Negative

A computed tomography (CT) scan of the thorax, abdomen, and pelvis was done to screen for a possible underlying malignancy, which was negative. A muscle biopsy was performed of the right thigh, which revealed evidence of skeletal muscle with inflammation and necrosis but with macrophagic involvement and minimal lymphocytic infiltrate, which was consistent with probable immune-mediated necrotizing myopathy (IMNM). She was treated with corticosteroids, and her CK level was reduced to 3041 U/L after one week. She was discharged on 40mg once daily of oral prednisolone for one month, and her dose was weaned by 10mg a month until 12.5mg was achieved. Her inflammatory markers had normalized four months after discharge. Her oral steroids were successfully weaned off 17 months after her admission. She was followed up regularly in the rheumatology outpatient department with regular blood tests monitoring her creatine kinase levels. She did not experience adrenal insufficiency following the cessation of steroids.

Two and a half years after her initial presentation, she was referred to the rheumatology outpatient clinic with increased difficulty climbing stairs and weight loss. Her symptoms were noticeably milder; she had full power of 5/5 in all four limbs with no evidence of synovitis or tenderness. Unfortunately, she was found to have been prescribed 40mg of atorvastatin in the community by her general practitioner for primary prevention of cardiovascular disease.

Her blood tests at the time, including full blood count, C-reactive protein, urea and electrolytes, anti-CCP antibodies, rheumatoid factor, haematinics, and thyroid stimulating hormone, were unremarkable. Her creatine kinase was elevated at 602 U/L (reference range 25-200 U/L). A repeat myositis antibody screen was negative. The atorvastatin was stopped, and she was started on 30 mg of oral prednisolone, which was weaned down over the course of two months. Her CK levels were reduced to 341 U/L at the end of this period. A repeat CT-thorax, abdomen, and pelvis was requested to re-assess for an underlying malignancy, which revealed a left renal parenchymal lesion that is currently being investigated by urology. Her CK levels had normalized seven months after the start of her second presentation, and her symptoms had resolved.

## Discussion

Statin-induced myopathy is a heterogeneous entity, of which immune-mediated necrotizing myopathy (IMNM) is the most severe form. This is the case of a patient who developed IMNM twice after being exposed to two separate statins, simvastatin and atorvastatin.

Immune-mediated necrotizing myopathy (IMNM) is an autoimmune inflammatory myopathy characterized by muscle weakness, positive autoantibodies, elevated creatine kinase levels, and evidence of inflammatory myopathy on muscle biopsy. IMNM is traditionally divided into three subtypes based on positive autoantibodies: anti-signal recognition particle (anti-SRP) positive autoantibodies, anti-hydroxy-3-methylglutaryl-CoA reductase (anti-HMGCR) positive autoantibodies, and antibody-negative IMNM [2].

Statin exposure is a risk factor for developing anti-HMGCR, up to 89% in certain population cohort studies [2]. This may be due to the increased expression of HMGCR seen with statin use or the concurrent binding of statins to HMGCR, which provides an immunogenic foci for the immune system to develop antibodies [2].

Patients classically present with proximal muscle weakness, and this may be the only symptom. Skin and extra-muscular manifestations can be seen in less than 10% of patients. Interstitial lung disease may be associated with up to 20% of patients. Malignancy can be associated with some inflammatory myopathies, such as dermatomyositis. With IMNM, this varies based on subtype; an increased risk is seen with antibody-negative IMNM, a weak risk with the anti-HMGCR subtype, and no malignancy association with the anti-SRP subtype [2].

Investigations would typically reveal an elevated creatine kinase (median 4700 IU/L), which correlates with the myofiber necrosis typically seen on biopsy. MRI of the affected muscle groups with T1-weighted and STIR can show prominent hyperintensities, which indicate muscle edema with inflammation or myofiber necrosis. Fatty replacement of muscle tissue on MRI imaging would be indicative of a chronic and severe myopathic process. MRI cannot reliably differentiate between the myopathic processes and thus has limited utility in the initial diagnosis of IMNM. In addition to muscle fiber necrosis, a muscle biopsy would also show upregulation of major histocompatibility complex class I, with macrophagic and some lymphocytic infiltration [2]. Other investigations can include an EMG to confirm the presence of a myopathic disease, and further imaging, such as a CT of the thorax, abdomen, and pelvis to investigate for an associated underlying malignancy, should be considered [3].

Treatment typically includes withdrawal of the statin, high-dose oral steroids at 1mg/kg/day or 0.5g-1g/day of IV steroids for 3-5 days initially and tapering down to the minimum dose. Second-line therapy options include methotrexate, rituximab, and intravenous immunoglobulins (IVIG) [4].

The learning point of this case is that IMNM can recur with the use of different statins, likely reflecting a class effect. Patients with prior statin-induced myopathy who have had a statin re-challenge were found to have altered skeletal muscle gene expressions, which may indicate a genetic predisposition towards developing myalgia and myositis [5].

## Conclusions

This case highlights the importance of avoiding the prescription of statins in patients with a prior history of statin-induced necrotizing myopathy, as it risks recurrence of symptoms and the associated morbidity of chronic immunosuppressant therapy to control their symptoms.
